# Biological Maturation, Body Morphology and Physical Performance in 8–16 year-old obese girls from Montes Claros – MG

**DOI:** 10.2478/hukin-2014-0102

**Published:** 2014-11-12

**Authors:** Alex S. Freitas, António J.B. Figueiredo, Andréia L.R. de Freitas, Vinícius D. Rodrigues, Alexandre A.C. da Cunha, Fernando F. Deusdará, Manuel J. Coelho e Silva

**Affiliations:** 1 Faculty of Sport Sciences and Physical Education - University of Coimbra; 1 Department of Physical Education and Sport - State University of Montes Claros - UNIMONTES.

**Keywords:** chronologic age, bone age, age at menarche, overweight and obesity

## Abstract

Measurements of maturity depend on the biological system considered since differences are often found in performance and body size in subjects of the same chronological age. The objective of this study was to identify associations between biological maturation, body morphology and physical performance in girls aged from 8.0 to 15.9 year-old and to verify the bone age in obese girls and compare it with chronological age. For that purpose 2040 (11.9 ± 2.3 years) school girls from Montes Claros, participated in this study. Regular anthropometric measures as height and body mass were taken. Triceps, biceps, subscapular, abdominal, suprailiac and calf skinfolds were also registered. Physical performance was assessed trough the test of a standing long jump, handgrip strength and 20 m multistage shuttle run. Maturational status, the average age at menarche and identification of PHV (maturity off set) were determined by means of the retrospective method. Girls with the BMI above the 95th percentile got their bone age evaluated through X-ray of the left hand/wrist, in accordance with the FELS method. It was possible to find an average age at menarche of 11.30 ± 0.70, while the average age at PHV was 12.17 ± 0.71 years of age. It was observed that both body composition and physical performance showed a tendency to increase with advancing age. However, when controlling the effect of maturation, despite having higher values in body composition the post-menarche girls group did not show higher levels of physical performance. In all age groups, obese girls showed mean rates of bone age higher than chronologic age (12.25 ± 2.09 and 14.09 ± 2.35, respectively, p=0.000). Chronological age should be used with caution when evaluating obese teenagers as it may underestimate biological age.

## Introduction

Biological maturation can be understood as a process that characterizes human growth and development, suffering individual variations in time and rate at which this process occurs (Malina et al., 2004; Cumming et al., 2008; Guedes, 2011). Therefore, it is important to note that transition from childhood to puberty is characterized not only by changes in sexual maturation, but also by changes in body composition and physical performance both in children and adolescents. Consequently, differences are often found in performance and body size in subjects of the same chronological age (Mirwald et al., 2002; Machado et al., 2009; Santos et al., 2011). In this sense Gouveia et al. (2009) reported that individuals with earlier maturation tended to have higher BMI values than their peers and that this perennial maturity had a long term effect on the increase in body fat, which may last into adulthood, thus increasing the risk of overweight and obesity

However, measurements of maturity depend on the biological system considered. Among all the most common indicators there is skeletal, sexual and somatic maturation (Malina et al., 2004; Rodrigues et al., 2010; Silva et al., 2010). Recognizing that the maturational process is a major factor in changes and adaptations occurring in body composition, fitness and physical performance in children and adolescents, the aim of this study was to identify possible associations between sexual maturation provided by the age at menarche, somatic maturation given by the age of peak height velocity (PHV), body composition and physical performance of girls aged from 8 to 16 years in the city of Montes Claros – MG. As a secondary objective we wanted to verify the bone age in obese girls and compare it with chronological age.

## Material and Methods

### Type of Study and Sample

This study is characterized as being descriptive, cross-sectional with quantitative data analysis. The group of participants was comprised of 2040 girls aged between 8.0 and 15.9 years (11.9 ± 2.3 years), randomly selected from 16 public and private schools from the city of Montes Claros. Parents were informed of the content of the experiment and signed an informed consent form allowing their children to participate. Data collection was conducted during physical education classes.

### Procedures

Anthropometric measurements were performed according to the parameters proposed by Lohman et al. (1988). Body mass (kg) was measured to the nearest 0.1 kg using a standard digital scale (Plena, Brazil). To evaluate body height (in cm) a precision stadiometer with a range scale of 0.1 cm was used (Sanny, Brazil). The following skinfolds, triceps, biceps, subscapular, abdominal, suprailiac and calf were measured with a precision skinfold caliper (Sanny, Brazil). From these measurements, the BMI and percentage of fat mass were established.

Physical performance was assessed trough the test of a standing long jump, handgrip strength (Lafayette hand dynamometer, Lafayette) and 20 m multistage shuttle run.

Regarding maturational status, the average age at menarche and identification of PHV (*maturity off set*) were determined by the retrospective method based on body weight, height, sitting height, length of the lower limbs and chronological age. The girls with the BMI above the 95th percentile (Kuczmarski et al., 2002) got their bone age evaluated through X-ray of the left hand/wrist, in accordance with the FELS method.

In the present study, “*on-time*” was defined as an age of PHV within ± 1 yr old from the mean age; “late” was defined as an age of PHV> 13.16; and “early” was defined as an age of PHV <11.6 years.

### Statistical Analysis

Normality assumptions were tested with the Shapiro-Wilk test. The independent samples *t* test was used to compare chronological age and bone age among the obese group. One way ANOVA was used to compare maturational status, body composition and physical performance. Multivariate analysis was use to verify the effect of maturational status on body composition and physical performance. Effect size was calculated by partial eta square (η^2^). All data were analyzed using SPSS for Windows 19.0 and α value was set at 0.05.

## Results

The results showed a trend toward an increase in values of body composition and physical performance with the advance of age. However, when this increase was controlled by maturational status, it is noteworthy that the girls who had gone through the advent of menarche had higher values with respect to body composition than their counterparts. In physical performance there were no differences in pre and post-menarche girls on any of the tests. In the age group of 14 years on the handgrip strength test mature girls presented higher values (p = 0.005) and girls with late maturation showed a higher PHV than girls with normal or advanced maturation.

The average age at menarche (11.30 ± 0.70 years) and age at PHV (12.17±0.71 years) were significantly different (p = 0,000).

**Figure 1:**
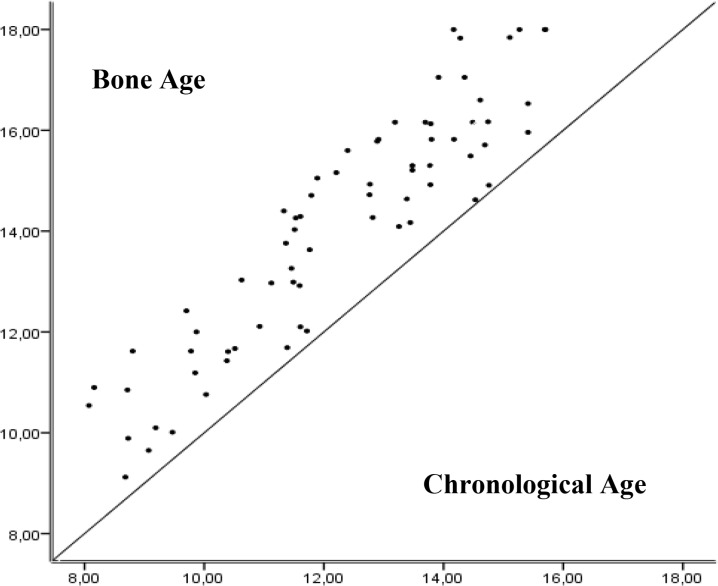
Differences between bone age and chronological age in obese girls

Regarding obese girls the trend was the same as for the rest of the group in all variables, however, the values of the BMI (27.15 ± 3.15) and fat mass (37.39 ± 7.35) were significantly different. In the same group, another interesting data was the variation in chronological age compared with bone age. In all age groups the obese girls had a mean bone age (14.09 ± 2.35) superior (p = 0.000) to the mean chronological age (12.25 ± 2.09) (Figure 1).

## Discussion

The primary objective of the present study was to identify possible associations between sexual maturation, somatic maturation, body composition and physical performance of girls aged from 8 to 16 years in the city of Montes Claros – MG.

According to the obtained data, a difference in the growth process after having distinguished the pre and post-menarche groups was observed. Female subjects who had not yet passed by the advent of menarche showed nonlinear growth with advancing age, therefore female subjects who were already mature presented an increase in all morphological variables in all age groups, exhibiting higher values in comparison with their counterparts. According to Malina et al. (2004), and Bergman et al. (2008), the pattern of physical growth is almost linear and nearly similar between subjects without significant changes until the onset of puberty, however, one may note some variability in growth at different ages in the body as a whole or in specific body parts. In fact, concerning body composition, Machado et al. (2011), in a study carried out in Brazil, found the same trend in physical growth with advancing age in girls aged from 10 to 14 years, especially in height, body mass and the BMI.

In terms of physical performance, the results showed that the strength levels increased with age and that from 14 years this improvement decreased and occasionally showed a plateau. As for cardiorespiratory fitness (shuttle run), pre-menarche groups always performed better than their post-menarche counterparts. It can be also noted that girls classified as late maturation, showed higher values in the tests of physical performance. It should be highlighted that post-menarche girls presented higher values of body fat, revealing that excessive fat accumulation may have negatively influenced the results of cardiorespiratory fitness. These facts have already been reported by Malina et al. (1995) who stated that fat mass exerted a negative influence on the cardiorespiratory fitness, motor performance and physical fitness. Another factor that may explain better results of the pre-menarche girls could be the declining levels of physical activity which occurred through teenage years, implying a consequent decline in physical performance and increased body mass. In this context, Sherar et al. (2010) and Rodrigues et al. (2010) reported that the growth spurt, changes in body composition and sexual maturation were influencing variables in this mechanism and that generally the biological maturation may be an important factor in sedentary behavior during adolescence. Moreover, Figueiredo et al. (2011) stated that maturation was directly linked to obesity in adolescents, being this trend more evident in girls.

Regarding handgrip strength, the data showed an increase in strength with increasing age, and there is a clear trend for post-menarche girls to report higher values than their pre-menarche peers. Corroborating our results, Silva and Oliveira (2010), in Sergipe, Brazil, found significant differences in the handgrip strength test between pre and post-menarche girls (17.8 ± 4.4 kg and 3.9 ± 21.5 kg; p <0.05, respectively). Furthermore, Malina et al. (2004) reported that post-menarche girls were stronger, and that earlier maturation girls tended to be stronger in early adolescence, being nevertheless these differences reduced with advancing age.

When we compared the average age at menarche to the age at PHV in the whole sample, we found values of 11.30 ± 0.70 years for menarche and 12.17 ± 0.71 years for the PHV, showing significant differences (p = 0.000), although Wellens et al. (1990) and Malina et al. (2004) reported that menarche occurred after PHV. However, the same authors also claimed that the age at menarche may vary among populations, as well as over time and that in well-nourished girls was regulated mainly by genetic factors. Interestingly, the age at menarche in the present study was lower than in other studies conducted in Brazil. For instance, Roman et al. (2009), in a study conducted in Cascavel, Brazil, with a sample of 2761 girls from 10 to 17 years, found an average age at menarche of 12.2 ± 1.2 years. Likewise, in their longitudinal study for over 20 years, Farias et al. (2012), in Ilhabela, Brazil, noticed a secular trend of declining age at menarche from 12.5 ± 1.5 to 12.2 ± 1.0 years old. In New York, USA, Terry et al. (2009) with the participation of 841 girls aged from 7 years, determined the average age at menarche from 12.5 ± 1.72 years. Even with the change in the mean age at menarche in the aforementioned studies, it seems consensual in the literature that this lower age at menarche appears to be directly associated with the risk of overweight and obesity. Having acknowledged that possibility, Ribeiro et al. (2006) in a study conducted in Portugal, found a positive association between excessive body fat and early sexual maturation.

As a secondary objective of the present study, we verified the bone age in girls who were classified as obese and compared it with chronological age.

When effect of the maturational status in the differences in body composition and physical performance was analyzed, it was noted that both the BMI and body fat presented higher values in the early maturity group (p=0.000), while the late maturation group presented better results in the hand grip strength and cardiorespiratory fitness. These results are similar to those reported by Coelho e Silva et al. (2013) using the prediction of the percentage of the mature height achieved, with Portuguese young people from the Azores (11–15 years), on which associations were found between advanced maturational status and higher rates of overweight and obesity. Such results are in accordance with the statements of Malina et al. (2004) and Gouveia et al. (2009), which pointed out that children and adolescents in early maturation were taller and heavier from the age of six than their counterparts with normal and late maturation. Thus, some indicators such as maturational age at PHV and the occurrence of early menarche have often been associated with increased adiposity (Alberga et al., 2012).

With respect to the group of girls classified as obese (Table 4), it can be noticed that besides the high levels in body composition variables and low performance on shuttle run and standing long jump tests, there was also a difference between chronological age and bone age (Figure 1). It was found that obese girls were advanced at maturation and according to Malina et al. (2004), when the bone age was advanced in over a year, this person should be classified as early maturing.

Comparatively, Machado and Barbanti (2007), using the method of Greulich and Pile to determine bone age, in a sample of 233 students from 9 to 13 years old, found differences in body composition associated with bone age with regard to which the girls with early maturation showed consistently higher values. The same way, Chonchaiya et al. (2008), analyzed 62 (38 boys and 24 girls) obese subjects from 5 to 17 years from Bangkok, Thailand, and found significant differences between chronological and biological age, pointing to an advancement of bone age in obese children and adolescents. In Madeira Island, Portugal, Gouveia et al. (2009) conducted a study with girls from 7 to 14 years of age and found alongside with a more advanced bone age higher values of the BMI.

This study faced some limitations as it was composed only by females and failed to take data from a longitudinal observation, nevertheless, it points to the existence of significant associations between biological maturation and their interactions with body composition and physical performance in children and adolescents.

## Conclusion

The average age at menarche was found to be different than the average age at PHV (11.30 ± 0.70 and 12.17 ± 0.71 years of age, respectively) for girls of Montes Claros, Brazil.

Given the results it was observed that both the body composition and physical performance showed a tendency to increase with advancing age. However, when controlling the effect of maturation, despite having higher values in body composition, the post-menarche girls group did not show higher levels of physical performance, except for the group of 14 years in handgrip strength.

Obese girls showed a trend of growth in all variables of body composition with advancing age, especially the BMI and fat mass. In the group of obese girls chronological age was different from bone age. Such results may point out to the existence of a relationship between obesity and biological maturation, which is accelerated by the advance of bone age and the premature onset of the pubertal changes.

Some caution should be addressed when using chronological age for evaluating young obese girls, as it may underestimate biological age.

## Figures and Tables

**Table 1:** Differences in body composition and physical performance in pre and post-menarche girls between 8.0 and 11.9 years

Variable	Menarch	8.0–8.9		9.0–9.9		10.0–10.9		11.0–11.9	

		Pre n=256	Post n=00	Pre n=254	Post n=01	Pre n=255	Post n=12	Pre n=165	Post n=86
Height (cm)	Pre	135.60±8.26		138.55±7.88		138.48±9.50		152.22±7.73	
	Post	---		141.50		143.65±11.14		153.70±7.52	
	
Body Mass (kg)	Pre	30.32±7.26		32.34±7.81		31.85±8.42		41.26±8.21	
	Post	---		46.00		37.69±14.62^*^		46.32±10.51^**^	
	
IMC	Pre	16.35±2.80		16.70±2.98		16.41±2.98		17.75±2.64	
	Post	---		22.97^*^		17.74±4.95		19.54±4.32^**^	
	
Body Fat (%)	Pre	22.56±7.62		23.08±8.26		21.36±6.47		21.51±5.80	
	Post	---		45.41^**^		24.21±11.05		25.75±8.28^**^	
	
Handgrip strength (kg)	Pre	11.79±2.84		12.62±3.38		13.14±4.17		20.58±4.32	
	Post	---		14.80		15.21±4.48		21.29±5.51	
	
Standing long jump (m)	Pre	107.22±24.31		101.87±23.60		106.46±26.18		120.57±22.37	
	Post	---		107.00		92.00±32.67^*^		118.14±24.40	
	
Shuttle-run (n^º^ turns)	Pre	14.96±6.02		18.05±7.22		15.54±7.30		22.36±10.54	
	Pos	---		12.00		13.75±7.31		21.90±10.65	

* p < 0.05

** p < 0.01

**Table 2:** Differences in body composition and physical performance in pre and post-menarche girls between 12.0 and 15.9 years.

Variables	Menarch	12.0–12.9		13.0–13.9		14.0–14.9		15.0–15.9	

		Pre n=77	Post n=176	Pre n=8	Post n=249	Pre n=01	Post n=250	Pre n=00	Post n=251
Height (cm)	Pre	155.46±6.49		157.08±6.98		150.40		---	
	Post	156.15±7.40		158.94±6.56		161.70±6.35		162.54±6.61	
	
Body Mass (kg)	Pre	43.70±8.20		42.56±5.44		39.50		---	
	Post	45.22±8.65		48.52±9.89		53.63±10.20		54.61±9.50	
	
IMC	Pre	17.95±2.89		17.27±2.13		17.46		---	
	Post	18.48±2.97		19.16±3.56		20.47±3.62		20.66±3.30	
	
Body Fat (%)	Pre	23.05±6.13		18.62±3.98		22.96		---	
	Post	23.03±6.58		24.63±7.12^*^		27.15±6.71		27.86±6.63	
	
Handgrip strength (kg)	Pre	21.76±4.16		24.70±4.79		11.30		---	
	Post	21.74±3.95		22.82±4.45		24.86±4.80^**^		24.93±4.63	
	
Standing long jump (m)	Pre	122.03±21.16		118.38±23.47		106.00		---	
	Post	126.05±25.13		117.04±23.83		120.98±25.05		119.63±27.48	
	
Shuttle-run (n^º^ turns)	Pre	25.14±11.50		25.00±8.64		14.00		---	
	Pos	24.24±13.46		24.17±13.38		26.54±11.26		26.12±11.29	

* p < 0.05

** p < 0.01

**Table 3:** Effect of maturational status on the change in body composition and physical performance

**Variável**	**Late maturity**	**Normal maturity**	**Early maturity**	**F**	**p**	**η^2^**
Height (cm)	153.61±9.44	150.16±13.46	150.37±9.65	410.53	0.000^**^	0.287
Body Mass (kg)	45.04±10.55	41.76±13.07	45.18±11.17	340.49	0.000^**^	0.251
BMI	18.86±3.13	18.09±3.58	19.83±3.78	121.02	0.000^**^	0.106
Body Fat (%)	24.87±6.88	23.65±7.26	27.71±8.61	68.90	0.000^**^	0.063
Handgrip (kg)	22.70±5.18	18.87±6.78	16.84±6.10	81.60	0.000^**^	0.074
Standing long jump (m)	125.25±25.41	114.35±25.53	106.39±27.26	2.30	0.101	0.002
Shuttle-run (n^º^ turns)	25.42±12.21	21.33±11.24	14.85±6.97	3.29	0.037^*^	0.003

* p < 0.05

** p < 0.01

**Table 4:** Descriptive values of chronological age, bone age, body composition and physical performance of obese girls

**Variables**	**N**	**Minimum**	**Maximum**	**Mean ± SD**
Chronological age (years)	70	8.07	15.70	12.25±2.09
Bone age (years)	70	9.12	18.00	14.09±2.35
Height (cm)	70	129.60	170.50	152.76±9.15
Body Mass (kg)	70	36.10	91.40	63.98±12.22
IMC	70	21.49	39.40	27.15±3.15
Body fat (%)	70	20.99	50.81	37.39±7.55
Σ Skinfolds (mm)	70	22.70	225.50	82.51±55.84
Handgrip strength (kg)	70	5.10	32.50	21.30±6.24
Standing long jump (m)	70	56.00	162.00	109.30±23.48
Shuttle-run (n^º^ turns)	70	1.00	46.00	17.17±9.88
